# Neoadjuvant pyrotinib, trastuzumab, and docetaxel for HER2-positive breast cancer (PHEDRA): a double-blind, randomized phase 3 trial

**DOI:** 10.1186/s12916-022-02708-3

**Published:** 2022-12-27

**Authors:** Jiong Wu, Zefei Jiang, Zhenzhen Liu, Benlong Yang, Hongjian Yang, Jinhai Tang, Kun Wang, Yunjiang Liu, Haibo Wang, Peifen Fu, Shuqun Zhang, Qiang Liu, Shusen Wang, Jian Huang, Chuan Wang, Shu Wang, Yongsheng Wang, Linlin Zhen, Xiaoyu Zhu, Fei Wu, Xiang Lin, Jianjun Zou

**Affiliations:** 1 Department of Breast Surgery, Fudan University Shanghai Cancer Center, No.270, Dong’an Road, Xuhui District, Shanghai, 200032 China; 2 Department of Medical Oncology, The Fifth Medical Center of Chinese PLA General Hospital, Beijing, China; 3 Department of Breast Disease, Henan Breast Cancer Center, The Affiliated Cancer Hospital of Zhengzhou University & Henan Cancer Hospital, Zhengzhou, China; 4 Breast Surgery, Zhejiang Cancer Hospital, Hangzhou, China; 5 Breast Surgery, Jiangsu Province Hospital, Nanjing, China; 6 Department of Breast Cancer, Cancer Center, Guangdong Provincial People’s Hospital, Guangdong Academy of Medical Sciences, Guangzhou, China; 7 Breast Center, The Fourth Hospital of Hebei Medical University, Shijiazhuang, China; 8 Breast Disease Center, The Affiliated Hospital of Qingdao University, Qingdao, China; 9 Breast Surgery, The First Affiliated Hospital Zhejiang University, Hangzhou, China; 10 Oncology Department, The Second Affiliated Hospital of Xi’an Jiaotong University, Xi’an, China; 11 Department of Breast Surgery, Sun Yat-Sen Memorial Hospital, Sun Yat-Sen University, Guangzhou, China; 12 Internal Medicine, Sun Yat-Sen University Cancer Center, Guangzhou, China; 13 Breast Surgery, The Second Affiliated Hospital, Zhejiang University, Hangzhou, China; 14 Department of Breast Surgery, Fujian Medical University Union Hospital, Fuzhou, China; 15 Department of Breast Surgery, Peking University People’s Hospital, Beijing, China; 16 Department of Breast, Shandong Cancer Hospital, Jinan, China; 17 Department of Thyroid and Breast Surgery, The Affiliated Huai’an No.1 People’s Hospital of Nanjing Medical University, Huai’an, China; 18 Clinical Research & Development, Jiangsu Hengrui Pharmaceuticals Co., Ltd, Shanghai, China

**Keywords:** Breast cancer, HER2, Pyrotinib, Neoadjuvant treatment, Phase 3

## Abstract

**Background:**

Pyrotinib (an irreversible pan-ErbB inhibitor) plus capecitabine has survival benefits and acceptable tolerability in patients with HER2-positive metastatic breast cancer. We further assessed addition of pyrotinib to trastuzumab and docetaxel in the neoadjuvant setting.

**Methods:**

In this multicenter, double-blind, phase 3 study (PHEDRA), treatment-naive women with HER2-positive early or locally advanced breast cancer were randomly assigned (1:1) to receive four neoadjuvant cycles of oral pyrotinib or placebo (400 mg) once daily, plus intravenous trastuzumab (8 mg/kg loading dose, followed by 6 mg/kg) and docetaxel (100 mg/m^2^) every 3 weeks. The primary endpoint was the total pathological complete response (tpCR; ypT0/is and ypN0) rate per independent central review.

**Results:**

Between Jul 23, 2018, and Jan 8, 2021, 355 patients were randomly assigned, 178 to the pyrotinib group and 177 to the placebo group. The majority of patients completed four cycles of neoadjuvant treatment as planned (92.7% and 97.7% in the pyrotinib and placebo groups, respectively). The tpCR rate was 41.0% (95% CI 34.0 to 48.4) in the pyrotinib group compared with 22.0% (95% CI 16.6 to 28.7) in the placebo group (difference, 19.0% [95% CI 9.5 to 28.4]; one-sided *P* < 0.0001). The objective response rate per investigator was 91.6% (95% CI 86.6 to 94.8) in the pyrotinib group and 81.9% (95% CI 75.6 to 86.9) in the placebo group after the neoadjuvant treatment, resulting in an increase of 9.7% (95% CI 2.7 to 16.6). The most common grade 3 or worse adverse events were diarrhea (79 [44.4%] in the pyrotinib group and nine [5.1%] in the placebo group), neutropenia (33 [18.5%] and 36 [20.3%]), and decreased white blood cell count (29 [16.3%] and 24 [13.6%]). No deaths were reported during neoadjuvant treatment.

**Conclusions:**

The primary endpoint of the study was met. Neoadjuvant pyrotinib, trastuzumab, and docetaxel significantly improved the tpCR rate compared with placebo, trastuzumab, and docetaxel, with manageable toxicity, providing a new option for HER2-positive early or locally advanced breast cancer.

**Trial registration:**

ClinicalTrials.gov, NCT03588091

**Supplementary Information:**

The online version contains supplementary material available at 10.1186/s12916-022-02708-3.

## Background

HER2 overexpression or gene amplification accounts for approximately 15 to 20% of all breast cancers [1]. For primary operable HER2-positive breast cancer, neoadjuvant anti-HER2 therapy has become a routine treatment strategy. The achievement of pathological complete response (pCR) after neoadjuvant therapy represents a well-established surrogate study endpoint for the long-term outcomes in terms of event-free survival (EFS) and overall survival, especially when defined as eradication of invasive tumor from both breast and lymph nodes [2, 3].

Humanized monoclonal antibody trastuzumab is the cornerstone of therapy for HER2-positive breast cancer. Despite the improvement in pCR rate and EFS, 15% of patients might relapse due to resistance to trastuzumab [4]. The proposed mechanisms might be involved with structural defects within the HER2 receptor, the constitutive activation of downstream elements, the activation of the downstream pathways by other members of the HER family, or intracellular alterations that affect the PI3K pathway [5, 6]. Therefore, adding a second anti-HER2 agent with trastuzumab-complementary activity represents a rationale strategy for neoadjuvant therapy [7].

Pertuzumab is a HER2-directed humanized monoclonal antibody with different binding sites to trastuzumab [8]. Until now, pertuzumab in combination with trastuzumab and chemotherapy remains the only approved dual anti-HER2 regimen in the neoadjuvant setting for patients with HER2-positive early breast cancer [9, 10]. Compared with monoclonal antibodies, tyrosine kinase inhibitors (TKIs) also show robust anti-tumor activities against breast cancer but have advantages of oral administration route, inhibition of both ligand-dependent and independent signaling, and low risk of cardiac toxicity.

Pyrotinib is an irreversible pan-ErbB inhibitor targeting EGFR/HER1, HER2, and HER4 [11]. Compared with lapatinib plus capecitabine, pyrotinib plus capecitabine exhibited an over 20% increase in objective response rate (ORR), a significant benefit in progression-free survival (median, 12.5 versus 6.8 months; hazard ratio [HR], 0.39), and a clear trend towards improvement in overall survival (median, not reached versus 26.9 months; HR, 0.69) in patients with pretreated metastatic HER2-positive breast cancer [12–14]. Combined with the findings of other pivotal studies [15–17], pyrotinib in combination with capecitabine was approved as the second-line standard-of-care for HER2-positive metastatic breast cancer in China. In this context, we conducted a phase 3 study to further investigate the efficacy and safety of adding pyrotinib to trastuzumab and docetaxel in the neoadjuvant setting.

## Methods

### Study design and patients

The PHEDRA study was a multicenter, double-blind, randomized, placebo-controlled, phase 3 study done at 17 hospitals in China (NCT03588091). Eligible patients were treatment-naive women aged 18 to 75 years with pathologically confirmed HER2-positive, early (T2 to 3, N0 to 1, M0) or locally advanced (T2 to 3, N2 to 3, M0) breast cancer with primary tumor larger than 2 cm in diameter. HER2 positivity was determined locally and defined as 3+ staining intensity by immunohistochemistry or *HER2* gene amplification by fluorescence in situ hybridization according to the 2013 American Society of Clinical Oncology/College of American Pathologists guidelines [18]. Other main inclusion criteria included Eastern Cooperative Oncology Group performance status of 0 or 1, known estrogen receptor (ER) and progesterone receptor (PR) status, and adequate hepatic, renal, bone marrow, and cardiac function based on laboratory assessments. For adequate cardiac function, baseline left ventricular ejection fraction (LVEF) of 55% or more as measured by echocardiography and Fridericia-corrected QT (QTcF) interval of less than 470 ms was required. Key exclusion criteria included metastatic disease (stage IV), inflammatory breast cancer, other malignancies, prior anti-cancer therapy or radiotherapy for any malignancy (except cured cervical carcinoma in situ, basal cell carcinoma, or squamous cell carcinoma), impaired cardiac function, uncontrolled hypertension, pregnancy, and refusal to use contraception.

The study protocol and all amendments were approved by the Ethics Committee of each study site. The study was conducted in accordance with the Declaration of Helsinki and Good Clinical Practice guidelines. All patients provided written informed consent.

### Randomization and masking

Patients were randomly assigned (1:1) to receive either pyrotinib, trastuzumab, plus docetaxel (pyrotinib group) or placebo, trastuzumab, plus docetaxel (placebo group). A stratified, permuted block randomization with a block size of four was performed, with stratification by primary tumor size (>2 cm and ≤5 cm versus >5 cm) and hormone receptor status (ER and/or PR positive versus ER and PR negative, the positivity cutoff for both was ≥1%). All investigators, patients, and the funder of the study were masked to treatment allocation.

### Interventions

Patients received oral pyrotinib 400 mg once daily or matched placebo in combination with intravenous trastuzumab (8 mg/kg loading dose, 6 mg/kg maintenance dose) and docetaxel (100 mg/m^2^) on day 1 of each 21-day cycle for four cycles, followed by surgery within 14 days (Additional file 1: Fig. S1). Thereafter, patients were given adjuvant therapy with three cycles of FEC (fluorouracil 500 mg/m^2^, epirubicin 100 mg/m^2^, and cyclophosphamide 500 mg/m^2^; all given intravenously every 3 weeks) and subsequent anti-cancer treatments at physicians’ discretion in accordance with clinical practice guidelines.

Primary prophylaxis for diarrhea was not prespecified. Based on the results of the interim analysis, the independent data monitoring committee (IDMC) recommended the implementation of proactive diarrhea management (PDM). Primary prophylaxis of neutropenia using a single, 6 mg fixed dose of mecapegfilgrastim on day 2 of each cycle was prespecified. Other granulocyte colony-stimulating factors were permitted if mecapegfilgrastim was intolerable or unavailable at the local study center.

### Assessments

To assess the tumor response to neoadjuvant therapy, patients underwent mammography, ultrasounds, and MRI at baseline and after completion of the neoadjuvant therapy (before surgery). Objective responses were assessed by investigators according to the Response Evaluation Criteria in Solid Tumors, version 1.1. Pathological response was assessed by local pathology review and masked independent central review using tumor tissue resection specimens obtained at surgery.

Laboratory assessments and vital signs were done at baseline, at each cycle during neoadjuvant therapy, before surgery, and at each cycle during adjuvant therapy. Cardiac monitoring was done with echocardiography at baseline, at every two cycles during neoadjuvant therapy, and on the day before adjuvant therapy and with 12-lead electrocardiograms at baseline, at every two cycles during neoadjuvant therapy, and before surgery. Adverse events were monitored continuously until 28 days after the last dose of FEC treatment and graded according to the Common Terminology Criteria for Adverse Events, version 4.03.

### Outcomes

The primary endpoint was the rate of total pathological complete response (tpCR), defined as the absence of any residual invasive cancer on hematoxylin and eosin staining of the resected breast specimen and all sampled ipsilateral lymph nodes (ypT0/is, ypN0) after neoadjuvant therapy and surgery, as assessed by independent central review. Protocol-defined secondary endpoints were tpCR rate per local pathology review, ORR (defined as the proportion of patients who had a best overall response of complete or partial response during neoadjuvant therapy), EFS (defined as the time from randomization to the first documentation of progressing disease while on study therapy, postoperative disease recurrence, or death from any cause), disease-free survival (DFS, time from surgery to the first documentation of postoperative disease recurrence or death from any cause), distant disease-free survival (DDFS, defined as the time from surgery to the first documentation of postoperative distant metastasis or death from any cause), and safety. Disease recurrence in EFS and DFS definitions referred to breast cancer recurrence, occurrence of second primary breast cancer, and occurrence of any other cancer. Exploratory endpoints included breast pathological complete response (bpCR) rate per local pathology review and independent central review. Results for EFS, DFS, and DDFS are not included in this report as the data are not mature.

### Statistical analysis

With 294 patients, the study had 85% power to detect an increase of 18% in tpCR rate (from 30% in the placebo group to 48% in the pyrotinib group), at a one-sided *α* level of 0.025, considering that 10% of enrolled patients would be unevaluable for pathological response assessment. Comparison between groups was done using the Cochran-Mantel-Haenszel test stratified by the randomization strata. One prespecified interim analysis was planned when pathological responses were available for 158 patients. As of Aug 30, 2019, the pathological responses were assessable in 159 patients, and interim analysis was done by an independent statistics team from KNOWLANDS MedPharm Consulting (Shanghai, China). The IDMC reviewed the results on Nov 6, 2019, and recommended continuing the trial with an increased sample size of 354 patients for final analysis (the criteria for IDMC recommendation described in Additional file 1: Supplementary Methods). Considering the sample size was increased, the Cui, Hung, and Wang method was used in the primary analysis for the tpCR rate in order to control the type I error [19]. A one-sided *P* value was reported, and the value of less than 0.025 was considered significant.

Efficacy analyses were done in the full-analysis set, including all patients who underwent randomization and received at least one dose of study treatment, with patients analyzed according to their randomized assignment. Safety analyses were done in all patients who received at least one dose of study treatment, with patients included according to their actual treatment. The 95% CIs for the pCR rate were calculated using the Wilson method. The 95% CI for the between-group difference was calculated using the Wald method. Patients without a recorded assessment of pCR were regarded as non-responders. Prespecified subgroup analyses of the tpCR rate per independent central review were also done. Statistical analyses were conducted using SAS version 9.4 and sample size re-estimation was done using East version 6.5.

## Results

### Patients

**Fig. 1:**
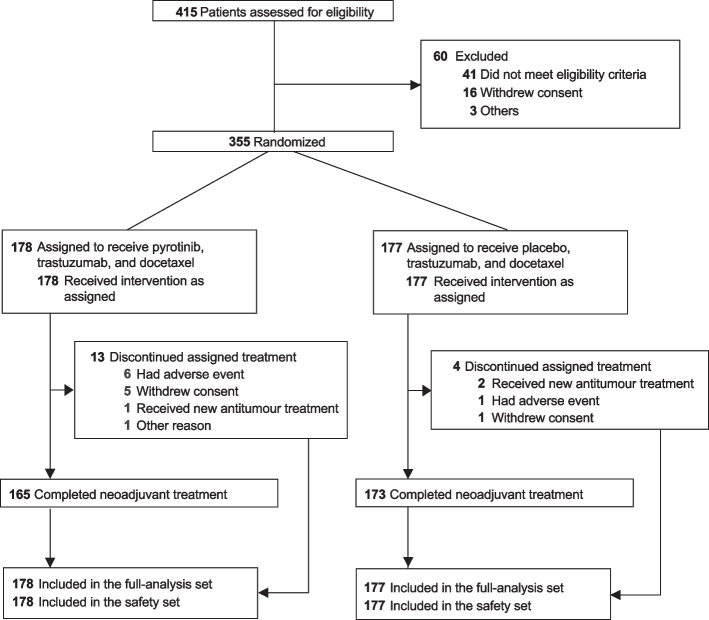
Trial profile

Between Jul 23, 2018, and Jan 8, 2021, 355 eligible patients were randomly assigned, 178 to receive pyrotinib, trastuzumab, and docetaxel and 177 to receive placebo, trastuzumab, and docetaxel (Fig. 1). The baseline characteristics were generally well balanced between the two groups (Table 1). The data cutoff date for the present analysis was Apr 30, 2021, which followed the last pathological assessment of enrolled patients.

**Table 1:** Baseline demographics and disease characteristics

	Pyrotinib, trastuzumab, and docetaxel (***n***=178)	Placebo, trastuzumab, and docetaxel (***n***=177)
**Age, years**		
Median (IQR)	50 (43 to 55)	50 (44 to 55)
≤40	37 (20.8%)	32 (18.1%)
41 to 64	130 (73.0%)	140 (79.1%)
≥65	11 (6.2%)	5 (2.8%)
**ECOG performance status**		
0	172 (97.2%)	172 (96.6%)
1	5 (2.8%)	6 (3.4%)
**Clinical status of lymph nodes**		
N0	41 (23.0%)	48 (27.1%)
N1	101 (56.7%)	93 (52.5%)
N2	27 (15.2%)	22 (12.4%)
N3	9 (5.1%)	14 (7.9%)
**Clinical stage**		
II	128 (71.9%)	125 (70.6%)
III	50 (28.1%)	52 (29.4%)
**Hormone receptor status**		
ER and/or PR positive	97 (54.5%)	98 (55.4%)
ER and PR negative	81 (45.5%)	79 (44.6%)
**Primary tumor size**		
>2 cm and ≤5 cm	156 (87.6%)	155 (87.6%)
>5 cm	22 (12.4%)	22 (12.4%)

Data are *n* (%), unless otherwise specified

*ECOG* Eastern Cooperative Oncology Group, *ER* estrogen receptor, *PR* progesterone receptor

All 355 patients received the study treatment. A total of 165 (92.7%) of 178 patients in the pyrotinib group and 173 (97.7%) of 177 patients in the placebo group completed four cycles of neoadjuvant treatment as planned (Fig. 1). The study treatment was discontinued in 13 (7.3%) patients in the pyrotinib group and four (2.3%) in the placebo group, mainly due to adverse events (6 [3.4%] and 1 [0.6%]), withdrawal of consent (5 [2.8%] and 1 [0.6%]), or start of new anti-cancer treatment (1 [0.6%] and 2 [1.1%]). No patients discontinued the neoadjuvant treatment because of disease progression. Five (2.8%) patients in the pyrotinib group and three (1.7%) in the placebo group who did not undergo surgery had no valid pathological assessment and were categorized as not achieving a pCR.

### Efficacy

**Fig. 2:**
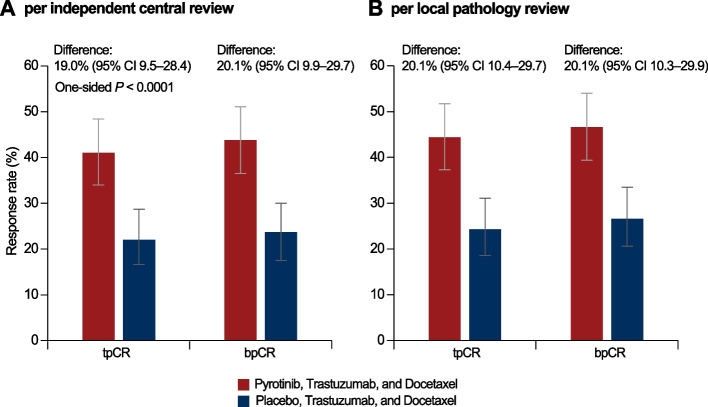
Total pathological complete response (tpCR) rate and breast pathological complete response (bpCR) rate. **A** Per independent central review; **B** per local pathology review. Patients with missing or unevaluable pCR status were considered non-responders. Error bars show 95% CIs for the pCR rate in each group, which were calculated using the Wilson method. Comparison between groups was done using the Cochran-Mantel-Haenszel test stratified by the randomization strata, and the 95% CI for the between-group difference was calculated using the Wald method

According to the independent central review, the tpCR (ypT0/is, ypN0) rate was significantly higher in the pyrotinib group than that in the placebo group (41.0% [95% CI 34.0 to 48.4] versus 22.0% [95% CI 16.6 to 28.7]; absolute difference, 19.0% [95% CI 9.5 to 28.4], one-sided *P* < 0.0001, Fig. 2A). Local pathologists assessed tpCR rate also indicated similar improvement (44.4% [95% CI 37.3 to 51.7] in the pyrotinib group versus 24.3% [95% CI 18.6 to 31.1] in the placebo group; absolute difference, 20.1% [95% CI 10.4 to 29.7], Fig. 2B). The rate of bpCR (ypT0/is) was higher in the pyrotinib group either according to independent central review (43.8% [95% CI 36.5 to 51.1] versus 23.7% [95% CI 17.5 to 30.0]; absolute difference, 20.1% [95% CI, 9.9 to 29.7]) or local pathology review (46.6% [95% CI 39.4 to 54.0] versus 26.6% [95% CI 20.6 to 33.5]; absolute difference, 20.1% [95% CI, 10.3 to 29.9], Fig. 2A, B).

**Fig. 3:**
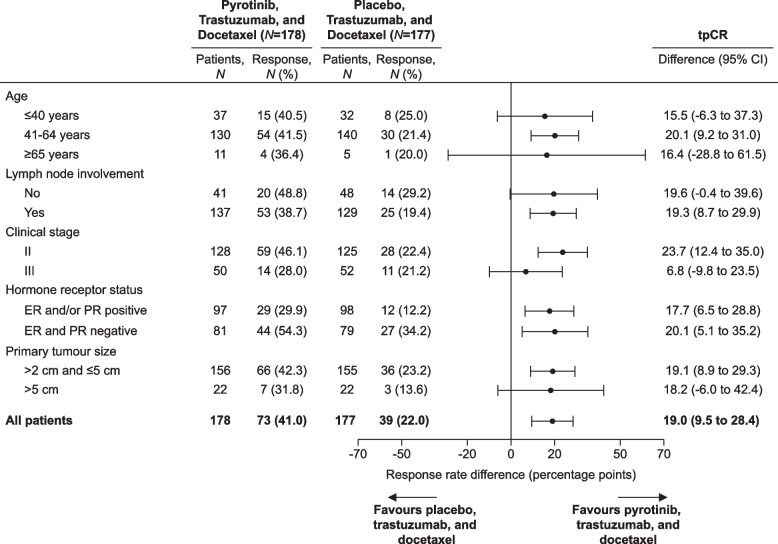
Subgroup analysis of total pathological complete response (tpCR) per independent central review. Differences between pyrotinib and placebo groups in each subgroup were shown, with the 95% CI being calculated using the Wald method. ER estrogen receptor, PR progesterone receptor

Consistent with the overall result, improvements in tpCR with pyrotinib, trastuzumab, and docetaxel were clearly observed across all predefined subgroups (Fig. 3).

The ORR per investigator was 91.6% (95% CI 86.6 to 94.8) in the pyrotinib group and 81.9% (95% CI 75.6 to 86.9) in the placebo group after the neoadjuvant treatment, resulting in an increase of 9.7% (95% CI 2.7 to 16.6, Additional file 1: Table S1). Complete responses were achieved by 28 (15.7%) of 178 patients in the pyrotinib group and 11 (6.2%) of 177 patients in the placebo group.

### Safety

Exposure of the individual components of neoadjuvant treatment is shown in Additional file 1: Table S2. A total of 127 (71.3%) patients in the pyrotinib group and 66 (37.3%) in the placebo group experienced adverse events of grade 3 or 4 (Table 2). The grade 3 or 4 adverse events occurring in at least 15% of patients in either group were diarrhea (79 [44.4%] in the pyrotinib group and nine [5.1%] in the placebo group), neutropenia (33 [18.5%] and 36 [20.3%]), and decreased white blood cell count (29 [16.3%] and 24 [13.6%]). Serious adverse events were reported in 26 (14.6%) patients in the pyrotinib group and 12 (6.8%) patients in the placebo group (Additional file 1: Table S3). No deaths occurred during the neoadjuvant treatment.

**Table 2:** Adverse events during neoadjuvant therapy occurring in ≥15% of patients in either group

	Pyrotinib, trastuzumab, and docetaxel (***n***=178)		Placebo, trastuzumab, and docetaxel (***n***=177)	
	Any grade	Grade 3 or 4	Any grade	Grade 3 or 4
Any adverse events	178 (100.0%)	127 (71.3%)	176 (99.4%)	66 (37.3%)
Diarrhea	178 (100.0%)	79 (44.4%)	93 (52.5%)	9 (5.1%)
Vomiting	131 (73.6%)	23 (12.9%)	42 (23.7%)	2 (1.1%)
Anemia	118 (66.3%)	11 (6.2%)	80 (45.2%)	2 (1.1%)
Alopecia	111 (62.4%)	0	147 (83.1%)	0
Nausea	85 (47.8%)	1 (0.6%)	51 (28.8%)	0
Asthenia	75 (42.1%)	0	70 (39.5%)	0
ALT increased	72 (40.4%)	5 (2.8%)	65 (36.7%)	5 (2.8%)
AST increased	66 (37.1%)	2 (1.1%)	58 (32.8%)	2 (1.1%)
WBC decreased	62 (34.8%)	29 (16.3%)	61 (34.5%)	24 (13.6%)
Weight decreased	59 (33.1%)	0	7 (4.0%)	0
Rash	58 (32.6%)	2 (1.1%)	32 (18.1%)	0
Neutropenia	57 (32.0%)	33 (18.5%)	54 (30.5%)	36 (20.3%)
Decreased appetite	55 (30.9%)	2 (1.1%)	32 (18.1%)	0
Bone pain	41 (23.0%)	0	51 (28.8%)	1 (0.6%)
Pain	39 (21.9%)	0	64 (36.2%)	0
Insomnia	39 (21.9%)	0	35 (19.8%)	0
Pyrexia	38 (21.3%)	2 (1.1%)	37 (20.9%)	0
PPE syndrome	37 (20.8%)	1 (0.6%)	50 (28.2%)	1 (0.6%)
Abdominal pain upper	35 (19.7%)	1 (0.6%)	14 (7.9%)	0
Stomatitis	33 (18.5%)	4 (2.2%)	10 (5.6%)	0
Hypokalemia	28 (15.7%)	9 (5.1%)	3 (1.7%)	0
Hypertriglyceridemia	26 (14.6%)	1 (0.6%)	41 (23.2%)	1 (0.6%)
Constipation	22 (12.4%)	0	45 (25.4%)	0
Cough	17 (9.6%)	0	30 (16.9%)	0

Data are *n* (%)

*ALT* alanine aminotransferase, *AST* aspartate aminotransferase, *WBC* white blood cell, *PPE* palmar-plantar erythrodysesthesia

The worst diarrhea in severity was grade 3. It mainly occurred in the first cycle and gradually decreased during treatment (Additional file 1: Fig. S2). In the pyrotinib group, the median time from the first dose to the onset of grade 3 diarrhea was 9 days, with a median duration of 2 days per event and a median cumulative duration of 5 days (Additional file 1: Table S4). Discontinuation of pyrotinib due to diarrhea was required in only one (0.6%) patient. Of the 178 patients in the pyrotinib group, 106 were recruited before the implementation of the PDM strategy, including the use of loperamide as the first choice of anti-diarrheal agents and strict application of loperamide recommended dose (4 mg initially and an additional 2 mg following each diarrhea stool, not exceeding 16 mg/day), and 72 were recruited after the implementation of PDM strategy (Additional file 1: Table S5). The incidence of grade 3 diarrhea after PDM implementation decreased to 36.1% (26/72), compared with 50.0% (53/106) before PDM implementation (cycle 1, 29.2% versus 44.3%; cycle 2, 10.1% versus 21.8%; cycle 3, 7.2% versus 14.1%; cycle 4, 4.5% versus 11.1%). The median cumulative duration of grade 3 diarrhea was shortened in patients enrolled after the PDM implementation (2 days versus 6 days).

Grade 3 neutropenia was reported in 15 (8.4%) versus 20 (11.3%) patients in the pyrotinib versus placebo group, and grade 4 neutropenia occurred in 18 (10.1%) versus 16 (9.0%) patients, respectively (Additional file 1: Table S6). Five (2.8%) patients in the pyrotinib group and two (1.1%) patients in the placebo group developed febrile neutropenia of any grade. Generally, grade 3 or 4 neutropenia, febrile neutropenia, and decreased white blood cell count mainly occurred during the first cycle of treatment in both pyrotinib and placebo groups and reduced in the second cycle and thereafter.

No major cardiac toxicities were observed in terms of changes in LVEF and QTcF values during treatment. No patients in the pyrotinib group and one (0.6%) in the placebo group had a LVEF of less than 50% and a decrease of 10% or more from baseline. Four (2.2%) patients in the pyrotinib and five (2.8%) in the placebo group had a QTcF of 480 ms or more and a change of at least 60 ms from baseline.

## Discussion

The PHEDRA trial met its primary endpoint, demonstrating that neoadjuvant pyrotinib, trastuzumab, and docetaxel achieved a significantly higher rate of tpCR than placebo, trastuzumab, and docetaxel (41.0% versus 22.0%; absolute difference, 19.0%, one-sided *P* < 0.0001) in patients with HER2-positive early or locally advanced breast cancer.

Similar to the studies of pertuzumab, we adopted 12 weeks of neoadjuvant therapy followed by surgery and adjuvant FEC therapy. In the NeoSphere international phase 2 study, pertuzumab plus trastuzumab and docetaxel showed a significantly improved bpCR compared with trastuzumab and docetaxel (45.8% versus 29.0%) [9]. The tpCR rate was also higher with pertuzumab plus trastuzumab and docetaxel (39.3% versus 21.5%) [9]. The PEONY study reported a consistent result in the tpCR rate in the Asian population (39.3% with pertuzumab plus trastuzumab and docetaxel versus 21.8% with placebo plus trastuzumab and docetaxel; difference, 17.5% [95% CI 6.9 to 28.0]) [10]. Our study indicated comparable results with the PEONY study, both in the dual HER2 blockade group and single blockade control group, suggesting that pyrotinib plus trastuzumab and docetaxel might be an alternative option for HER2-positive breast cancer in the neoadjuvant setting.

Several clinical trials compared neoadjuvant therapy with a TKI plus trastuzumab-based regimen versus trastuzumab-based regimen [20–26]. Although all studies reported a numeric increase in bpCR or tpCR rate, only the CHER-LOB and NeoALTTO studies reached statistical significance. Differences in chemotherapy regimen, administration sequence of chemotherapy and anti-HER2 drugs, and duration of neoadjuvant therapy (16 to 26 weeks) might contribute to the inconsistent findings. In the CHER-LOB study, lapatinib and trastuzumab were administered for 26 weeks (throughout a sequential regimen of 12-week paclitaxel followed by 12-week FEC after 2 weeks of interval) [20]. The tpCR was 46.7% with lapatinib plus trastuzumab and chemotherapy versus 25% with trastuzumab and chemotherapy. In the NeoALTTO study, lapatinib and trastuzumab were administered for 18 weeks with paclitaxel being started at week 6 [25]. The tpCR rate was 46.8% with lapatinib and trastuzumab plus chemotherapy versus 27.6% with trastuzumab plus chemotherapy. These data, both in the dual HER2 blockade group and single blockade control group, were relatively higher in contrast to those reported in studies of pertuzumab and this study of pyrotinib, which might be caused by longer duration of neoadjuvant therapy with trastuzumab with or without lapatinib. Of note, only 60.5% of patients in the lapatinib plus trastuzumab and chemotherapy group completed planned neoadjuvant treatment, compared with 91.9% of patients in the trastuzumab and chemotherapy group [25]. In our study, 92.7% of patients completed the 12-week neoadjuvant pyrotinib plus trastuzumab and docetaxel as planned, suggesting that this neoadjuvant regimen represented a new effective option with short duration of neoadjuvant therapy and high compliance.

The most common severe toxicity with neoadjuvant pyrotinib plus trastuzumab and docetaxel was consistent with the known safety profile of individual components. Diarrhea in the pyrotinib group was characterized by mild or moderate severity, early onset, and short duration. Generally, diarrhea was manageable with anti-diarrheal agents, and only one patient discontinued pyrotinib due to diarrhea. Of note, implementation of the PDM strategy obviously reduced the incidence of grade 3 diarrhea in each cycle of the neoadjuvant therapy and shortened the cumulative duration of grade 3 diarrhea. Thus, when diarrhea occurs in clinical practice, loperamide at recommended dose should be given followed by close follow-up or observation.

When docetaxel at a dose of 75 mg/m^2^ was used, in either dual or single HER2 blockade groups, 45 to 57% of patients in the NeoSphere study and 32.7 to 38.1% of patients in the PEONY study suffered grade 3 or worse neutropenia, and 7 to 8% of patients in the NeoSphere study suffered febrile neutropenia. Mecapegfilgrastim is a long-acting recombinant human G-CSF with the advantage of once-per-cycle dosing and convenient dose management [27]. In our study, 100 mg/m^2^ docetaxel was used, and the majority of patients (82.0%) received 6 mg fixed dose of mecapegfilgrastim as primary prophylaxis for neoadjuvant chemotherapy-induced neutropenia. Grade 3 or 4 neutropenia occurred in 18.5% of patients in the pyrotinib group and 20.3% of patients in the placebo group, and febrile neutropenia occurred in 2.8% and 1.1% of patients, respectively. These results indicated that the addition of pyrotinib did not increase the incidence of neutropenia, and primary prophylaxis for neutropenia using mecapegfilgrastim was effective.

This study had some limitations. First, placebo, trastuzumab, and docetaxel was chosen as the control, because pertuzumab was not approved as a component of neoadjuvant therapy in China at the study design. Second, this report was for the final analysis of the primary endpoint tpCR; survival data were immature. The study is still ongoing and the data would be reported in the future. In addition, a phase 3 study of pyrotinib versus placebo after trastuzumab-based adjuvant therapy in HER2-positive breast cancer (NCT03980054) is being conducted to investigate whether the positive results in the neoadjuvant setting would be substantiated in the adjuvant setting in terms of invasive DFS.

## Conclusions

Overall, pyrotinib, trastuzumab, and docetaxel as neoadjuvant treatment achieved a statistically significant and clinically meaningful improvement in the tpCR rate for patients with HER2-positive early or locally advanced breast cancer compared with placebo, trastuzumab, and docetaxel, with an acceptable safety profile, supporting the approval of this combination as a new neoadjuvant treatment option in China. To our knowledge, this is the first phase 3 study supporting 12-week neoadjuvant treatment with a HER2-directed small-molecule TKI plus trastuzumab and chemotherapy in breast cancer patients.

## Supplementary Information

**Additional file 1: Figure S1.** Study diagram. **Figure S2.** Incidence of grade 3 diarrhea during neoadjuvant treatment. **Table S1.** Clinical responses following neoadjuvant treatment assessed by local investigator per RECIST v1.1. **Table S2.** Exposure of the individual components of study treatment. **Table S3.** Serious adverse events during neoadjuvant therapy. **Table S4.** Summary of data on diarrhea during neoadjuvant treatment. **Table S5.** Characteristics of diarrhea before and after proactive management. **Table S6.** Overview of neutropenia, febrile neutropenia, and decreased white blood cell count during neoadjuvant treatment period.

## Data Availability

Clinical data may be requested 24 months after study completion. Qualified researchers should submit a proposal to the corresponding author outlining the reasons for requiring the data. The leading clinical site and sponsor will check whether the request is subject to any intellectual property restriction. The use of data must also comply with the requirements of the Human Genetics Resources Administration of China and other country- or region-specific regulations. A signed data access agreement with the sponsor is required before accessing shared data.

## Abbreviations

bpCR: Breast pathological complete response
DDFS: Distant disease-free survival
DFS: Disease-free survival
ECOG: Eastern Cooperative Oncology Group
EFS: Event-free survival
ER: Estrogen receptor
IDMC: Independent data monitoring committee
LVEF: Left ventricular ejection fraction
ORR: Objective response rate
pCR: Pathological complete response
PDM: Proactive diarrhea management
PR: Progesterone receptor
QTcF: Fridericia-corrected QT
TKI: Tyrosine kinase inhibitor
tpCR: Total pathological complete response
