# Supplementary material for: Neoadjuvant pyrotinib, trastuzumab, and docetaxel for HER2-positive breast cancer (PHEDRA): a double-blind, randomized phase 3 trial
Source: BMC Med. 2022 Dec 27;20:498. doi: 10.1186/s12916-022-02708-3 (PMC9795751; doi:10.1186/s12916-022-02708-3)
Supplement: Supplementary file 1 — Additional file 1: Figure S1. Study diagram. Figure S2. Incidence of grade 3 diarrhea during neoadjuvant treatment. Table S1. Clinical responses following neoadjuvant treatment assessed by local investigator per RECIST v1.1. Table S2. Exposure of the individual components of study treatment. Table S3. Serious adverse events during neoadjuvant therapy. Table S4. Summary of data on diarrhea during neoadjuvant treatment. Table S5. Characteristics of diarrhea before and after proactive management. Table S6. Overview of neutropenia, febrile neutropenia, and decreased white blood cell count during neoadjuvant treatment period. [file 12916_2022_2708_MOESM1_ESM.docx]

**Table of contents**

[Figure S1: Study diagram 2](#_Toc117861264)

[Figure S2: Incidence of grade 3 diarrhea during neoadjuvant treatment 3](#_Toc117861265)

[Table S1: Clinical responses following neoadjuvant treatment assessed by local investigator per RECIST v1.1 4](#_Toc117861266)

[Table S2: Exposure of the individual components of study treatment 5](#_Toc117861267)

[Table S3: Serious adverse events during neoadjuvant therapy 6](#_Toc117861268)

[Table S4: Summary of data on diarrhea during neoadjuvant treatment 7](#_Toc117861269)

[Table S5: Characteristics of diarrhea before and after proactive management 8](#_Toc117861270)

[Table S6: Overview of neutropenia, febrile neutropenia, and decreased white blood cell count during neoadjuvant treatment period 9](#_Toc117861271)

[Supplementary Methods 10](#_Toc117861272)

#

# Figure S1: Study diagram


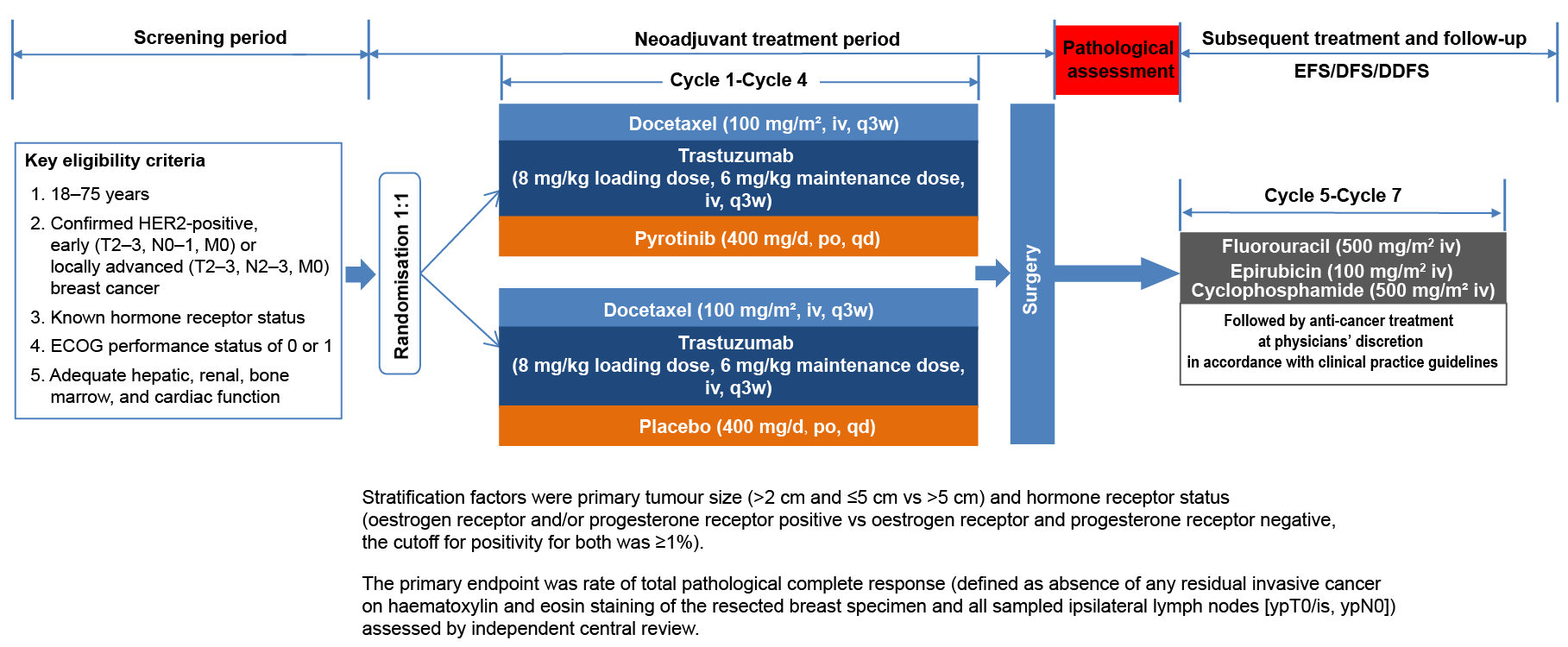


EFS=event-free survival; DFS=disease-free survival; DDFS=distant disease-free survival; ECOG=Eastern Cooperative Oncology Group; HER2=human epidermal growth factor receptor 2; q3w=every 3 weeks; qd=every day.

#

# Figure S2: Incidence of grade 3 diarrhea during neoadjuvant treatment


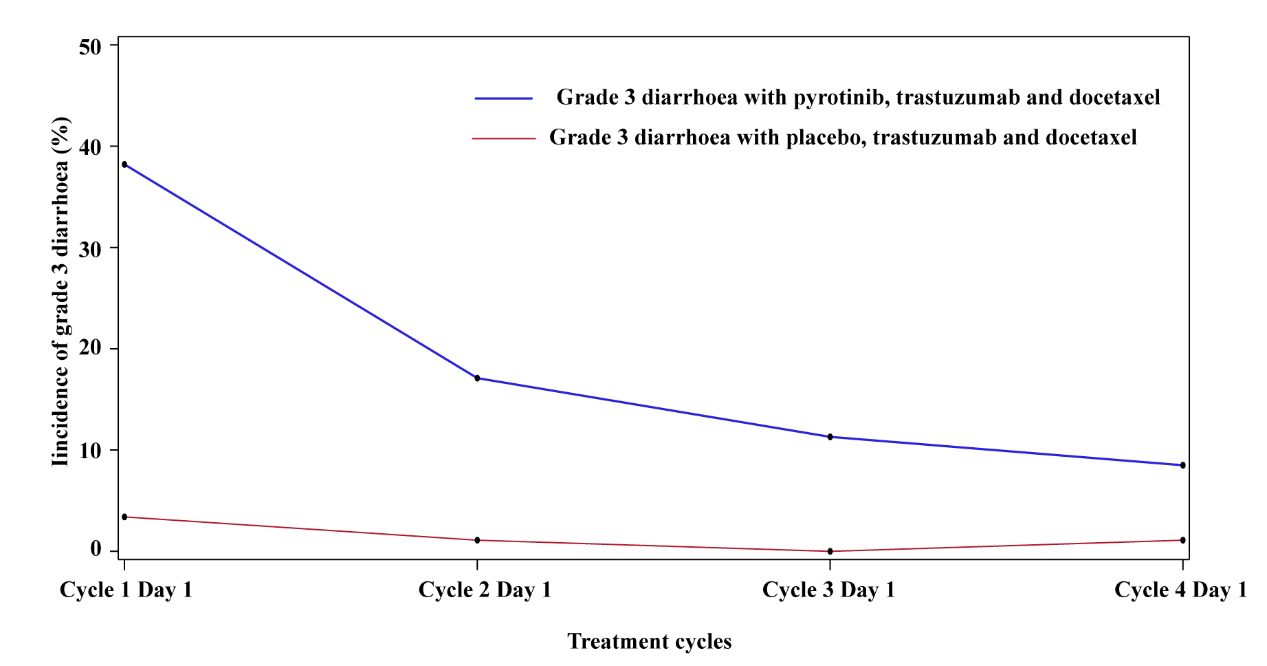


# Table S1: Clinical responses following neoadjuvant treatment assessed by local investigator per RECIST v1.1

|  | **Pyrotinib, trastuzumab, and docetaxel (n=178)** | **Placebo, trastuzumab, and docetaxel (n=177)** |
| --- | --- | --- |
| **Best overall response, n (%)** | | |
| Complete response | 28 (15.7%) | 11 (6.2%) |
| Partial response | 135 (75.8%) | 134 (75.7%) |
| Stable disease | 7 (3.9%) | 27 (15.3%) |
| Progressive disease | 0 | 1 (0.6%) |
| Not evaluable^*^ | 4 (2.2%) | 4 (2.3%) |
| No assessable^†^ | 4 (2.2%) | 0 |
| **Objective response rate** | | |
| n (%; 95% CI) | 163 (91.6%; 86.6–94.8) | 145 (81.9%; 75.6–86.9) |
| Between-group difference, % (95% CI) | 9.7% (2.7–16.6) | |

^*^Patient had no measurable target lesions at baseline. ^†^Patients who did not have a tumour response evaluation at post-baseline.

Objective response rate was defined as the proportion of patients achieving a complete response or partial response as the best tumour response during the neoadjuvant period. No confirmation was required for objective response.

# Table S2: Exposure of the individual components of study treatment

|  | **Pyrotinib, trastuzumab, and docetaxel**  **(n=178)** | **Placebo, trastuzumab, and docetaxel**  **(n=177)** |
| --- | --- | --- |
| **Pyrotinib/placebo** | | |
| Treatment duration, weeks |  |  |
| Median (range) | 12.3 (1–15) | 12.1 (0–15) |
| Number of cycles |  |  |
| Median (range) | 4 (1–4) | 4 (1–4) |
| Number of administration interruption |  |  |
| Number of patients, n (%) | 94 (52.8%) | 21 (11.9%) |
| Median (range) | 2 (1.0–8.0) | 1 (1.0–2.0) |
| Number of administration modifications |  |  |
| Number of patients, n (%) | 48 (27.0%) | 3 (1.7%) |
| Median (range) | 1 (1.0–2.0) | 1 (1.0–1.0) |
| Cumulative dose, mg |  |  |
| Mean (SD) | 30448.5 (7404.9) | 33831.0 (3366.7) |
| Median (range) | 33200  (2800–41200) | 33600  (800–42400) |
| **Trastuzumab** | | |
| Number of cycles |  |  |
| Median (range) | 4 (1–4) | 4 (1–4) |
| Number of infusion delay |  |  |
| Number of patients, n (%) | 29 (16.3%) | 14 (7.9%) |
| Median (range) | 1 (1.0–2.0) | 1 (1.0–2.0) |
| Cumulative dose, mg |  |  |
| Mean (SD) | 1514.4 (333.7) | 1586.8 (268.4) |
| Median (range) | 1534  (400–2652) | 1560  (464–2300) |
| **Docetaxel** | | |
| Number of cycles |  |  |
| Median (range) | 4 (1–4) | 4 (1–4) |
| Number of infusion delay |  |  |
| Number of patients, n (%) | 29 (16.3%) | 14 (7.9%) |
| Median (range) | 1 (1.0–2.0) | 1 (1.0–2.0) |
| Number of infusion modifications |  |  |
| Number of patients, n (%) | 9 (5.1%) | 2 (1.1%) |
| Median (range) | 1 (1.0–1.0) | 1 (1.0–1.0) |
| Cumulative dose, mg |  |  |
| Mean (SD) | 613.8 (115.3) | 639.3 (75.5) |
| Median (range) | 637 (146–800) | 640 (162–772) |

# Table S3: Serious adverse events during neoadjuvant therapy

|  | **Pyrotinib, trastuzumab, and docetaxel (n=178)** | | | **Placebo, trastuzumab, and docetaxel (n=177)** | | |
| --- | --- | --- | --- | --- | --- | --- |
|  | **Any grade** | **Grade 3** | **Grade 4** | **Any grade** | **Grade 3** | **Grade 4** |
| Any serious adverse event, n (%) | 26 (14.6%) | 14 (7.9%) | 5 (2.8%) | 12 (6.8%) | 8 (4.5%) | 0 |
| Pneumonitis | 4 (2.2%) | 3 (1.7%) | 0 | 0 | 0 | 0 |
| Hypokalemia | 4 (2.2%) | 2 (1.1%) | 2 (1.1%) | 0 | 0 | 0 |
| Alanine aminotransferase increased | 4 (2.2%) | 2 (1.1%) | 0 | 1 (0.6%) | 1 (0.6%) | 0 |
| Aspartate aminotransferase increased | 3 (1.7%) | 2 (1.1%) | 0 | 1 (0.6%) | 0 | 0 |
| Pyrexia | 3 (1.7%) | 1 (0.6%) | 0 | 0 | 0 | 0 |
| Vomiting | 2 (1.1%) | 1 (0.6%) | 0 | 1 (0.6%) | 1 (0.6%) | 0 |
| Diarrhea | 2 (1.1%) | 0 | 0 | 0 | 0 | 0 |
| Hemorrhoids | 1 (0.6%) | 1 (0.6%) | 0 | 1 (0.6%) | 1 (0.6%) | 0 |
| Febrile neutropenia | 1 (0.6%) | 1 (0.6%) | 0 | 1 (0.6%) | 1 (0.6%) | 0 |
| White blood cell count decreased | 1 (0.6%) | 1 (0.6%) | 0 | 0 | 0 | 0 |
| Pancreatitis | 1 (0.6%) | 1 (0.6%) | 0 | 0 | 0 | 0 |
| Appendicitis | 1 (0.6%) | 1 (0.6%) | 0 | 0 | 0 | 0 |
| Anemia | 1 (0.6%) | 1 (0.6%) | 0 | 0 | 0 | 0 |
| Hypotension | 1 (0.6%) | 1 (0.6%) | 0 | 0 | 0 | 0 |
| Syncope | 1 (0.6%) | 1 (0.6%) | 0 | 0 | 0 | 0 |
| Platelet count decreased | 1 (0.6%) | 0 | 1 (0.6%) | 0 | 0 | 0 |
| Bone marrow failure | 1 (0.6%) | 0 | 1 (0.6%) | 0 | 0 | 0 |
| Liver injury | 1 (0.6%) | 0 | 1 (0.6%) | 0 | 0 | 0 |
| Venous thrombosis limb | 1 (0.6%) | 0 | 0 | 1 (0.6%) | 0 | 0 |
| Decreased appetite | 1 (0.6%) | 0 | 0 | 0 | 0 | 0 |
| Urinary tract infection | 1 (0.6%) | 0 | 0 | 0 | 0 | 0 |
| Postoperative wound infection | 1 (0.6%) | 0 | 0 | 0 | 0 | 0 |
| Cholecystitis | 1 (0.6%) | 0 | 0 | 0 | 0 | 0 |
| Muscle twitching | 1 (0.6%) | 0 | 0 | 0 | 0 | 0 |
| Pneumonia | 0 | 0 | 0 | 2 (1.1%) | 1 (0.6%) | 0 |
| Abdominal pain | 0 | 0 | 0 | 1 (0.6%) | 1 (0.6%) | 0 |
| Device related infection | 0 | 0 | 0 | 1 (0.6%) | 1 (0.6%) | 0 |
| Postoperative wound complication | 0 | 0 | 0 | 1 (0.6%) | 1 (0.6%) | 0 |
| Influenza like illness | 0 | 0 | 0 | 1 (0.6%) | 0 | 0 |
| Palpitations | 0 | 0 | 0 | 1 (0.6%) | 0 | 0 |

#

# Table S4: Summary of data on diarrhea during neoadjuvant treatment

|  | **Pyrotinib, trastuzumab, and docetaxel**  **(n=178)** | **Placebo, trastuzumab, and docetaxel**  **(n=177)** |
| --- | --- | --- |
| **Diarrhea incidence n (%)** | | |
| All grades | 178 (100.0%) | 93 (52.5%) |
| Grade 1 | 19 (10.7%) | 60 (33.9%) |
| Grade 2 | 80 (44.9%) | 24 (13.6%) |
| Grade 3 | 79 (44.4%) | 9 (5.1%) |
| Cycle 1 | 68 (38.2%) | 6 (3.4%) |
| Cycle 2 | 29 (16.3%) | 2 (1.1%) |
| Cycle 3 | 19 (10.7%) | 0 |
| Cycle 4 | 14 (7.9%) | 2 (1.1%) |
| Grade 4 or 5 | 0 | 0 |
| **Median time from first dose to onset (IQR), days** | | |
| All grades | 3 (2–5) | 7 (4–16) |
| Grade 3 | 9 (5–12) | 16 (7–23) |
| **Median duration per diarrhea episodey (IQR), days** | | |
| All grades | 3 (2–7) | 2 (1–4) |
| Grade 3 | 2 (2–3) | 2 (1–2) |
| **Having diarrhea more than 3 times, n (%)** | | |
| All grades | 149 (83.7%) | 26 (14.7%) |
| Grade 3 | 15 (8.4%) | 0 |
| **Median cumulative duration (IQR), days** | | |
| Grade 3 | 5 (2–8) | 2 (1–2) |
| **Median time since the first onset to recovery (IQR), days** | | |
| All grades | 5 (2–12) | 2 (2–4) |
| **Treatment or dose modification for pyrotinib or placebo due to diarrhea, n (%)** | | |
| Treatment interruption | 66 (37.1%) | 4 (2.3%) |
| Dose reduction after treatment interruption | 26 (14.6%) | 0 |
| Dose reduction | 5 (2.8%) | 0 |
| Treatment discontinuation | 1 (0.6%) | 0 |

# Table S5: Characteristics of diarrhea before and after proactive management

|  | **Before the implementation of PDM** | | **After the implementation of PDM** | |
| --- | --- | --- | --- | --- |
|  | **Pyrotinib, trastuzumab, and docetaxel (n=106)** | **Placebo, trastuzumab, and docetaxel (n=106)** | **Pyrotinib, trastuzumab, and docetaxel (n=72)** | **Placebo, trastuzumab, and docetaxel (n=71)** |
| **Diarrhea incidence, n (%)** | | | | |
| All grades | 106 (100.0%) | 57 (53.8%) | 72 (100.0%) | 36 (50.7%) |
| Grade 1 | 12 (11.3%) | 32 (30.2%) | 7 (9.7%) | 28 (39.4%) |
| Grade 2 | 41 (38.7%) | 18 (17.0%) | 39 (54.2%) | 6 (8.5%) |
| Grade 3 | 53 (50.0%) | 7 (6.6%) | 26 (36.1%) | 2 (2.8%) |
| Cycle 1 | 47 (44.3%) | 4 (3.8%) | 21 (29.2%) | 2 (2.8%) |
| Cycle 2 | 22 (21.8%) | 2 (1.9%) | 7 (10.1%) | 0 |
| Cycle 3 | 14 (14.1%) | 0 | 5 (7.2%) | 0 |
| Cycle 4 | 11 (11.1%) | 2 (1.9%) | 3 (4.5%) | 0 |
| Grade 4 or 5 | 0 | 0 | 0 | 0 |
| **Median time to the first onset (IQR), days** | | | | |
| All grades | 4 (2–5) | 7 (4–28) | 3 (2–4) | 6 (5–12) |
| Grade 3 | 9 (5–11) | 16 (7–24) | 9 (6–12) | 11 (6–16) |
| **Median duration per diarrhea episode (IQR), days** | | | | |
| All grades | 4 (2–9) | 2 (2–4) | 2 (1–5) | 2 (1–3) |
| Grade 3 | 2 (2–3) | 2 (2–2) | 2 (1–2) | 1 (1–1) |
| **Median cumulative duration (IQR), days** | | | | |
| Grade 3 | 6 (3–9) | 2 (2–3) | 2 (2–5) | 1 (1–1) |
| **Median time since the first onset to recovery (IQR), days** | | | | |
| All grades | 7 (3–12) | 2 (2–4) | 3 (1–10) | 2 (1–4) |

PDM=proactive diarrhea management

# Table S6: Overview of neutropenia, febrile neutropenia, and decreased white blood cell count during neoadjuvant treatment period

|  | **Pyrotinib, trastuzumab, and docetaxel (n=178)** | **Placebo, trastuzumab, and docetaxel (n=177)** |
| --- | --- | --- |
| **Neutropenia, n (%)** | | |
| Any grade | 57 (32.0%) | 54 (30.5%) |
| Grade 1 | 6 (3.4%) | 5 (2.8%) |
| Grade 2 | 18 (10.1%) | 13 (7.3%) |
| Grade 3 | 15 (8.4%) | 20 (11.3%) |
| Grade 4 | 18 (10.1%) | 16 (9.0%) |
| Median time to first onset (IQR), days | 7 (6–63) | 6 (6–49) |
| Median duration per grade ≥3 neutropenia (range), days | 3 (1–16) | 3 (2–12) |
| Median cumulative duration of grade ≥3 neutropenia (range), days | 4 (2–16) | 3 (2–14) |
| **Febrile neutropenia, n (%)** | 5 (2.8%) | 2 (1.1%) |
| **Grade 3 or higher neutropenia, n (%)^*^** | | |
| Cycle 1 | 24 (13.5%) | 28 (15.8%) |
| Cycle 2 | 10 (5.9%) | 7 (4.0%) |
| Cycle 3 | 3 (1.8%) | 6 (3.4%) |
| Cycle 4 | 4 (2.4%) | 3 (1.7%) |
| **Grade 3 or higher febrile neutropenia, n (%)^*^** | | |
| Cycle 1 | 2 (1.1%) | 2 (1.1%) |
| Cycle 2 | 2 (1.2%) | 0 |
| Cycle 3 | 0 | 0 |
| Cycle 4 | 0 | 0 |
| **Grade 3 or higher white blood cell count decreased, n (%)^*^** | | |
| Cycle 1 | 20 (11.2%) | 20 (11.3%) |
| Cycle 2 | 8 (4.7%) | 2 (1.1%) |
| Cycle 3 | 2 (1.2%) | 1 (0.6%) |
| Cycle 4 | 4 (2.4%) | 2 (1.1%) |

***^*^***The denominator indicates number of patients with mecapegfilgrastim for prophylaxis use in this cycle.

# Supplementary Methods

This study included one interim analysis and a final analysis. The prespecified interim analysis was planned to be done by an independent statistics team from KNOWLANDS MedPharm Consulting (Shanghai, China) when pathological responses were available for 158 patients, with a futility boundary calculated using Lan-DeMets spending function with O’Brien-Fleming flavor-LD. The treatment assignment and interim analysis results would remain blinded to sponsor teams.

After reviewing the interim analysis results, the independent data monitoring committee would recommend early stopping for futility, trial continuation as planned, or trial continuation with an increase in sample size, based on the conditional power. If the conditional power obtained in the interim analysis was less than 9.1%, the study would be terminated early. If the conditional power was 30% to 80%, the study would continue with adjusted sample size. The maximum sample size allowed in this study was 1.5 times the originally planned sample size, and the power should be no more than 90% after adjustment.
